# Carry-Over of Aflatoxin B1 from Feed to Cow Milk—A Review

**DOI:** 10.3390/toxins15030195

**Published:** 2023-03-04

**Authors:** Andrea Zentai, Ákos Jóźwiak, Miklós Süth, Zsuzsa Farkas

**Affiliations:** National Laboratory of Infectious Animal Diseases, Antimicrobial Resistance, Veterinary Public Health and Food Chain Safety, University of Veterinary Medicine Budapest, 1078 Budapest, Hungary

**Keywords:** aflatoxin, carry-over, milk, cow, AFB1, AFM1

## Abstract

The conversion of aflatoxin B1 in feed consumed by cows into aflatoxin M1 in their milk poses a challenge to food safety, with milk being a popular staple food and because of the harmful effects of these compounds. This study aimed at reviewing scientific information about the extent of carry-over of AFB1 from feed to milk. A range of studies reported about correlations of carry-over with different factors, particularly with milk yield and AFB1 intake. The extent of carry-over considerably varies, being 1–2% on average, which may be as high as 6% in the case of increased milk production. Specific factors influencing transfer rates, including milk yield, somatic cell counts, aflatoxin B1 intake, source of contamination, seasonal effects, particle size of feed, and the effects of certain interventions, namely vaccination and the use of adsorbents, are identified as the most important and are discussed in this review. The different mathematical formulas describing carry-over and instances of their application are reviewed as well. These carry-over equations may lead to largely different results, and no single carry-over equation can be suggested as the best one. While the exact quantification of carry-over is difficult as the process depends on many factors, including individual variabilities between animals, the intake of aflatoxin B1 and milk yield seem to be the most important factors influencing the excreted amount of aflatoxin M1 and the rate of carry-over.

## 1. Introduction

Mycotoxins consumed by food-producing animals with their feed can be transferred into their tissues and occur in foods made of them for human consumption, e.g., in milk consumed daily. Aflatoxins are mycotoxins of great concern, with aflatoxin B1 (AFB1) produced by fungi on certain crops and converted into its metabolite aflatoxin M1 (AFM1) when ingested by cows with feed. AFB1 and AFM1 are both toxic and carcinogenic compounds. 

Quantification of this transfer (carry-over) provides information about the relationship of the levels of contamination in feed and the resulting contamination in milk. Several studies have been published in the last few decades to determine the carry-over of aflatoxins from feed to milk. This transfer can be described, on the one hand, by the so-called bioconcentration factor (BF), showing AFM1 concentration in milk as a percentage of the AFB1 concentration in feed (1).

(1)  BF=AFM1 concentration in milkAFB1 concentration in feed

Carry-over, on the other hand, is calculated with daily amounts, i.e., it indicates the percentage of consumed AFB1 with feed being eliminated in milk during a given day (2).

(2) $$Carry-over\;\%=\frac{m_{milk\;}\times c_{AFM1}}{m_{feed\;}\times c_{AFB1}}\times 100\;\%$$

where *m_milk_* and *m_feed_* are quantities of daily milk yield (kg) and the daily consumption of the feed (kg) contaminated with AFB1, and *c_AFM1_* and *c_AFB1_* indicate the concentrations of AFM1 and AFB1 in milk and in feed (μg/kg) [1].

Patterson et al. [2] studied the transfer of low levels of aflatoxins in six Friesian cows using thin-layer chromatographic means in 1980 and concluded that the latter formula could be regarded a more consistent index of carry-over than the ratio of toxin concentrations in feed and milk. 

Although cow milk is of primary significance regarding human health due to its high intake and wide variety of derived products, several studies have been conducted on the carry-over in other species (goats, sheep, buffalo, camel). Battacone et al. (2005) [3], for example, conducted detailed studies regarding carry-over into sheep milk, and they reported values of 0.26–0.33% [3]. Other authors reported 0.018–3.1% and 0.11–0.3% for carry-over in goats [4,5]; 0.2%, 2.13%, 3.13%, and 4.14–5.06% carry-over values were reported for buffalos [6,7,8,9]. In the following sections, the focus will be on the carry-over of aflatoxins into cow milk. 

The aim of this study was to review the published literature regarding the process and extent of AFB1 in feed transferred into AFM1 in cow milk and the most important factors influencing the abundance of carry-over. All articles found in the Scopus database were screened and relevant information regarding the extent of carry-over and the potential factors affecting this value were extracted from the publications. 

## 2. Aflatoxin in the Cow’s Body

A certain part of AFB1 consumed by ruminants via feed is degraded by the rumen microbiota and transformed into aflatoxicol (AFL) [10]. The remaining AFB1 is absorbed in the gut, first by passive diffusion in the small intestine, passing into the blood, where it is transported by red blood cells and plasma proteins to the liver.

Several aflatoxin metabolites are produced in the liver. In fact, they have been used as biomarkers of animal exposure, particularly for the evaluation of the effect of mycotoxin-reducing interventions, the decreasing residue level in the liver demonstrating the efficiency of the adsorbent studied [10]. The main hydroxylated metabolite of AFB1 is AFM1, which is formed through mediation of the cytochrome P450 (Cyt P450) enzyme system in the liver, and finally shows up in blood, milk, tissues, and biological fluids [10,11].

In fact, the oxidation of AFB1 into AFB1-8,9-epoxide occurs in the liver, which can react with RNA and DNA or with liver proteins, thus leading to hepatocellular carcinomas or liver toxicity. The epoxide is then converted into less toxic metabolites by hydroxylation, such as AFM1, aflatoxin Q1 (AFQ1), or aflatoxin P1 (AFP1) [12].

Other metabolites formed and excreted with milk, in addition to AFM1, include aflatoxin M2, a similar metabolite of aflatoxin B2, and aflatoxin M4, another hydroxylated metabolite of AFB1. Both AFM2 and AFM4 appear in milk at much lower concentrations than AFM1; therefore, they are considered as less in terms of public health significance [13].

AFB1 itself has been detected in heat-treated samples by some authors, suggesting that the AFB1 was not completely metabolized to AFM1. Moreover, other AFB1 metabolites have also been detected (e.g., AFM2) in heat-treated samples [10].

Ruminants are considered less susceptible to the harmful effects of mycotoxins than monogastric animals because of the activities of the rumen microbiota. The detoxification capacity of microbes, however, can become saturated, resulting in AFM1 accumulation in milk. Based on a few *in vivo* studies, AFM1 and aflatoxin Q1 (AFQ1) were the main metabolites detected in urine, feces, and milk; however, differences exist between species [10]. 

Experience from the carry-over studies [1,14,15,16,17,18,19,20,21,22,23,24] shows that aflatoxin consumed with contaminated feed appears relatively quickly (within a few hours after consumption) in milk as AFM1, reaches its maximum within a few days, and its level quickly falls when the source of contamination is eliminated from feed.

Frobish et al. (1986) [14], for example, studied the carry-over of aflatoxin into milk on 32 Holstein cows by feeding them cottonseed contaminated with aflatoxins. AFM1 occurred in milk 12 h after consumption of contaminated feed, reached steady-state conditions after 24 h, and its level decreased below the Food and Drug Administration action level of 0.5 µg/l within 24 h after removal of the contaminated feed [14].

Diaz et al. (2004) [15] experienced the occurrence of AFM1 in the first milk samples of cows receiving contaminated feed, and a maximum was reached after 3 days. Aflatoxin was eliminated from milk 4 days after the removal of toxin from the feed [15].

Masoero et al. (2007) [16] found that AFM1 appeared in milk immediately after the first AFB1 intake and continued to increase until it reached its plateau 5 days after the start of intake. The plateau of transfer (steady-state) was reached later compared to previous studies [16].

Xiong et al. (2015) [17] found no detectable AFM1 toxin in milk 3 days after stopping AFB1 intake, regardless of the AFB1 concentration in feed [17].

Guo et al. (2021) [1] studied the AFB1 metabolism in five lactating Holstein cows. The cows—being in 30–32 weeks of calving—were fed AFB1-contaminated feed (as total mixed ration) at 4 μg/kg of body weight for 13 days. After a 30-day withdrawal period, a higher single dose of 40 μg/kg of body weight was given to the animals to investigate the in vivo kinetics of AFB1 metabolism. After a second 30-day withdrawal period, another single dose of 40 μg/kg of body weight was given to the animals, and then they were sacrificed after 6 h. AFM1 occurred within 48 h of the repetitive AFB1 intake, and its elimination from milk lasted no longer than 2 days. After a single intake of the high dose, the AFM1 level in milk quickly increased and reached its peak after 24 h [1].

Maximum AFB1 concentration in blood was reached 35.0 ± 10.2 min after ingestion and then slowly decreased, with a half-life of 931.1 ± 30.8 min. Maximum AFM1 level was reached 4 h after intake. Its concentration then quickly decreased and was not detectable in milk after 96 h [1].

A range of values have been reported for the occurrence and elimination times of AFM1 in milk. This variability can be explained by the different sources of AFB1 contamination used in the studies (e.g., pure AFB1, naturally contaminated corn, cottonseed, or ground peanut meal), different contamination levels, and individual variability in the animals [1].

Table 1 summarizes the different occurrence times for AFM1 peak and AFM1 elimination times published in the literature. 

## 3. Aflatoxin B1-M1 Carry-Over Rates

Reported aflatoxin carry-over rates into cow milk vary to a great extent. EFSA estimated a 1–2% transfer rate of AFB1 from feed to AFM1 in milk, although this can increase to even 6% in the case of high-productivity cattle [13]. 

In some works, the bioconcentration factor, i.e., the ratio of AFB1 in feed to AFM1 in milk (Equation (1)), was estimated as a measure of carry-over in cows. Price et al. (1985) [25] investigated the average ratios of AFB1 level in feed and AFM1 level in milk during steady state, which was approximately 70 to 1. When the averages for the initial and the final periods were removed from the calculations, the refined ratio was 78 to 1. This ratio was lower than the approximately 300 to 1 cited by a review of previously published experiments [25].

Sumantri et al. (2012) [20] found significantly lower levels of AFM1 in milk in their investigation of low-level intakes. Carry-over was significantly lower (0.1%) as well compared to other studies. They noted that relatively less information was available regarding the potential adaptation of cows to detoxify AFB1 in the case of long-term exposure to AFB1-contaminated feeds. 

Stroud (2006) reviewed 14 studies investigating carry-over, based on which he concluded on an average transfer rate of 1%, when diets were dosed with up to 150 μg of aflatoxin/kg [21].

Bervis et al. (2021) [26] found an average carry-over of 3.22%, and when matching the positive milk samples with the contaminated feeds consumed, the calculated average carry-over value (8.79%) exceeded the 6% threshold established by EFSA for high-yielding cows.

AFB1-AFM1 carry-over rates found in the published literature are summarized in Table 2. 

## 4. Main Factors Influencing Aflatoxin Carry-Over

Several factors may play a role in carry-over [11,16,22,26,51,52,53]. Factors related to the feed include the level of AFB1 contamination, the quantity and characteristics of the feed consumed (e.g., the ratio of concentrated feed in the diet), or the geographical origin of the feed and the harvest time of the ingredients. 

Among the factors related to the animal, milk yield, phase of milk production (lactation stage), species, breed, time of day, general condition of the animal, metabolic status, rumen microflora, the microsomal mixed-function oxidase (MFO) activity, and hepatic biotransformation ability were reported, as well as the health status of the udder and the integrity of the alveolar (milk-producing) cell membranes of the udder, the interaction of toxins in the animal, changes in the blood–milk barrier and the rate of aflatoxin intake and digestion [11,16,22,26,51,52,53]. In the case of high-yielding cows, changes in the plasma–milk barrier and the significantly higher consumption of concentrated feeds might result in a higher carry-over rate [13].

Other factors affecting carry-over include the weather, the geographical location of the farm, and different animal feeding practices [11,16,22,26,51,52,53]. 

The most relevant factors are discussed in the following few sections. 

### 4.1. Stage of Lactation and Milk Yield

Research has shown a direct relationship between the extent of carry-over and milk yield and, thus, the stage of lactation. Milk yield is highest at the beginning of lactation (2–4 weeks) and, similarly, the transfer rate is higher at the beginning than in late lactation (34–36 weeks after calving) [22]. Carry-over in cows at the beginning of lactation might be 3.3–3.5-times greater than in later stages [28,54].

High milk yield is a phenotypic trait obtained as a result of breeding for increased milk production, but it is also influenced by intensive animal husbandry and feeding and is directly related to the stage of lactation. Newly calved cows quickly reach the peak of milk production, after which the production gradually decreases with increasing length of lactation [22].

Frobish et al. (1986) [14] noted a lack of correlation between the aflatoxin concentration in feed and the transfer rate, while the level of milk production impacted carry-over. Independently of AFB1 intake, high-producing cows had a higher percentage of total AFB1 transferred to AFM1 than low-producing cows. Mean carry-over for high producers was 2.14% (1.75–2.53%) while it was 1.35% (0.96–1.74%) for low producers.

Another important finding of this work was that milk production had no effect on the concentration of AFM1 in milk. Mean concentrations of AFM1 in high- and low-producing groups were 0.87 and 0.85 µg/l, respectively, while the group of high-producing cows produced twice the amount of milk and consumed only 50% more AFB1 than the low-producing group. The authors reasoned that the greater percentage of total AFB1 secreted as AFM1 by the high-producing group was due to the increased efficiency of AFB1-AFM1 conversion. They found no direct correlation between the level of milk production and the AFM1 concentration in milk [14].

Focusing on the effects of milk yield and AFB1 intake, Veldman et al. (1992) [28] conducted two experiments to study the AFM1 content in milk of cows given naturally contaminated feed with low levels of AFB1 during the early and later stages of milk production. 

In experiment 2, among cows receiving the same AFB1 intake, carry-over was higher in those with high milk yield than those with low yields. At a fixed AFB1 intake, the AFM1 concentration in milk seemed to be higher for cows with low milk yields, which was an opposite result to that of experiment 1, where cows at the beginning of lactation had higher AFM1 levels in their milk [28].

According to their results, individual differences could be observed in the excreted AFM1 quantities in milk between cows of the same stage of lactation, despite having similar AFB1 intakes. The authors stated that the carry-over of aflatoxins was independent of the AFB1 intake, which was in agreement with the conclusion of other authors observed at a higher intake range [28]. 

They also stated that the difference in milk AFM1 levels according to the milk yield observed in experiments 1 and 2 might be due to differences in the enzyme activities of the MFO system. They explained that the MFO system could convert AFB1 toxin not only to AFM1 toxin, but also to other hydroxylated derivatives (e.g., AFQ1 and AFP1), depending on the enzyme activities. Therefore, the conversion of AFB1-AFM1 by the MFO system may account for AFB1 carry-over variability.

According to the authors, the contradicting results between experiments 1 and 2 were in line with the conclusion of Van Egmond (1983) [55], that there was no relationship between AFM1 concentration in milk and milk yield. They stated that the increased permeability of the udder at the beginning of lactation could also explain the higher AFM1 content in milk of cows in lactation weeks 2–4, but other factors than animal factors, such as the precision of analytical methods, must also be taken into account.

In addition to the contradicting results of the two experiments, the results of experiment 1 are not consistent with the conclusions of some other carry-over studies as well [56].

Masoero et al. (2007) [16] investigated the effects of milk yield and somatic cell count, as a factor indicating udder inflammatory processes, on the aflatoxin carry-over in dairy cows. Thus, 34 Holstein cows participated in the study, which were grouped based on a preliminary experiment according to low (LY) and high milk yield (HY) (limit: 30 kg of milk per day), as well as low (LSCC) and high somatic (HSCC) cell count (limit: 350,000). They found AFM1 appearing in milk and being eliminated quickly. Carry-over values at the steady state were 2.32, 2.70, 1.48, and 1.29% for groups HY-HSCC, HY-LSCC, LY-HSCC, and LY-LSCC, respectively. A significant effect of milk yield was observed, while no relationship with somatic cell count level was found according to the results [16]. In the early stage of increase in the AFM1 plateau, there were differences between the individual experimental groups, which leveled off more and more as they approached the steady state. In the initial phase, a correlation with the number of somatic cells was observed, but only for the high-yield groups on the first two days of intake. These results suggested that high yield could amplify the effect of somatic cell count on carry-over [16].

Britzi et al. (2013) [22] studied carry-over of aflatoxins on 12 Israeli Holstein cows, which were grouped according to lactation stage, i.e., milk yield of cows in the middle of lactation (8–20 weeks after calving) was 36–51 kg, while it was 26–33 kg for those in late lactation (33–46 weeks after calving). Animals were milked three times a day, characteristic to intensive dairy industry. Average carry-over was 2.5% for cows in late and 5.8% for those in the middle stage of lactation [22]. 

Churchill (2017 and 2016) [39,41] studied the carry-over of aflatoxins on 36 Holstein cows in early and middle lactation stages. They explained that current regulations were based on older studies performed on low-yield cows (10–20 kg of milk daily), opposite to modern large-scale milk production depending on cows with 30–40 kg daily milk yields and three daily milkings. Carry-over was higher based on observations of cows with high milk yields. Applying linear regression on the experimental results, a carry-over value of 6.5 µg/100 µg was directly derived. The value of 6.5% justified that the transfer value for high-yielding cows was greater than the 1–2% previously set for low-producing cows, and AFM1 content in milk of high-yield cows may exceed the legal limit [39,41]. 

Costamagna and co-authors (2019) [45] studied the extent of carry-over on 36 cows. Cows were divided into three groups based on their lactation stage (90 days or less for high production, 90–150 days for medium production, and 150 days or more for low milk production). Average carry-over was 0.84% (0.05–5.93%). Milk yield had an impact on carry-over, regardless of lactation stage. The transfer rate was 1.21% (0.23–5.93%) for high-milk-yield cows and 0.48% (0.05–2.12%) for low-milk-yield cows.

These results confirm that in the initial stages of milk production and in proportion to the milk yield, the transfer of aflatoxins is higher than at a lower milk yield and in the later stages of milk production. Mathematical formulas established to describe the correlation of carry-over and milk yield provide additional insights into this relationship, which will be presented in Section 5. 

### 4.2. Somatic Cell Count

Some authors have highlighted that the infection of the udder increases the transfer of AFM1 into milk [28]. Lafont et al. (1980) [57] demonstrated correlation between carry-over and somatic cell counts (SCCs) independently of milk yield. Mastitis increases milk somatic cell counts, changes the composition of milk, and may impact AFM1 transfer due to increasing membrane permeability [16]. It must be noted here that SCC alone is just an indicator of animal health status, and it may vary due to various other factors. Masoero and coauthors (2007) [16] studied the effect of somatic cell counts on carry-over. In their work mentioned above, in addition to observing a significant impact of milk yield, no correlation with somatic cell counts was found. According to their conclusion, increased permeability of mammary gland due to inflammatory processes was not an explanation in itself for increased carry-over. Their results suggested, however, that high milk yield may intensify the effect of somatic cell counts on carry-over.

Neither Britzi et al. (2013) [22] nor Costamagna et al. (2019) [45] found correlation between carry-over and udder health expressed as somatic cell counts.

It is, therefore, not confirmed that there is a correlation between the somatic cell count and the carry-over of aflatoxins.

### 4.3. Effect of Seasons

It is reported that AFM1 levels are lower in the milk of animals fed by grazing than those fed compound feed and/or stored feed [54]. According to Flores-Flores et al. (2015) [54], some studies have reported higher levels of AFM1 in cow’s milk than in the milk of other animals (e.g., water buffalo, camel, sheep, and goat). These authors stated that the reason for this could be that the latter animals were mainly fed by grazing [54].

Virdis and co-authors (2014) [4], for example, stated that AFM1 contamination in milk from goats and sheep was generally lower than in cow’s milk, resulting from the fact that the former species was mainly fed by grazing and experienced less exposure to AFs through concentrate feeding. 

On the other hand, it is well known that warm and humid climates are favorable for the growth of aflatoxin-producing fungi; however, not all studies are in agreement regarding seasonal influence. 

It was reported by Patterson et al. (1980) [2] that the peak of aflatoxin M1 concentration in milk was in winter, when the level of concentrate feeding was highest. 

Spanish authors Hernández-Martínez and Navarro (2014) [51] investigated the occurrence of aflatoxins in the feed of dairy cows, as well as the potential impact of seasonal, geographical, and animal feeding factors on the degree of contamination. They estimated the exposure of dairy cows to AFB1 and, based on the theoretical intake, the extent of AFM1 carry-over. Regarding seasonal variation in AFB1 contamination, it was found to reflect the AFM1 levels in cow’s milk reported by Gómez-Arranz (2008) [58] for the study region (highest levels were measured in spring, followed by winter, summer, and autumn).

Signorini et al. (2012) [59] found higher mycotoxin contamination in milk during autumn than in spring. They explained their findings by the difference in diet composition in each season, with a higher ratio of ingredients more susceptible to mycotoxigenic fungi, i.e., concentrated feeds and corn silage during the autumn and less in spring. The ratios of these items in the total diet of dairy cattle in autumn were 57.26% and 65.71%, respectively, while they accounted for 22.22–40.9% of the diet in spring. It was estimated that the proportion of AFB1 intake from corn silage and concentrated feeds reached 66% of AFB1 in the diet during autumn, while it was only 48.18% during spring, which was explained by the differences in diet composition according to the season. 

Schirone et al. (2015) [60] found that mean concentrations in raw milk samples collected in autumn were higher than in other seasons, although no significant differences were observed between months. They hypothesized that such variation may be a result of toxin accumulation during storage. 

Costamagna and co-authors [45] concluded, in their above-mentioned study (2019), that the presence and concentration of AFM1 in milk were independent of seasons and lactation stage. Opposite to results reported by Signorini et al. (2012) [59], neither seasons nor milk yield and the amount of consumed feed could be associated with aflatoxin levels measured in milk. 

Khaneghahi Abyaneh et al. (2009) [61] found that the average AFM1 content in raw milk samples taken from dry (arid and semiarid) climates was significantly lower than in other climates, while the frequency of contamination in a dry climate was higher than in (semi)arid areas.

Pena-Rodas et al. (2018) [62] found, in their study, that the occurrence of AFM1 was 16.5% higher in drought years than in non-drought years. Consequently, the ratio of samples exceeding the regulatory limit was also higher. The number of samples positive to AFM1 was significantly correlated with the average annual temperature. They stated that their findings indicated that the prevalence of aflatoxin-positive samples increased with the intensity of drought and the annual average temperature. 

Further studies on AFM1 in milk along with records on weather conditions (temperature, humidity, rainfall, etc.) and conditions of storage could add more clarity to conclusions regarding the seasonal effects on carry-over and milk AFM1 content. 

### 4.4. AFB1 Level in Feed and AFB1 Intake

Price et al. (1985) [25] reported a linear positive relationship between the intake of AFB1 and the quantity of AFM1 excreted in cow milk. However, a linear relationship is not confirmed by other studies. 

Several authors reported that the carry-over rate of aflatoxin from feed to milk was independent of the concentration in feed. Veldman et al. (1992) [28] reported that carry-over was independent of AFB1 intake but was positively related to milk yield. The impact of milk yield on carry-over was further confirmed by other authors [14,16,22,39,45], as described in Section 4.1. 

Völkel and co-authors [52] reported, in their review, that the doses administered to animals have no direct influence on carry-over; however, the absolute amount excreted as AFM1 depends on the amount of AFB1 ingested [52].

The concentration of aflatoxin in milk, on the other hand, seems to be impacted by the concentration of the contaminant to be ingested by the animal. Frobish et al. (1986) [14] found a linear positive relationship between the AFB1 concentration in feed and the AFM1 concentration in milk and observed that AFB1 content in feed had no effect on carry-over. The authors found no direct relationship between milk yield and the concentration of AFM1 in milk [14]. 

Based on Veldman et al. (1992) [28], Van Eijkeren et al. (2006) [56] arrived at the conclusion that carry-over was positively correlated with milk production, AFM1 level in milk was inversely correlated to milk production at a fixed AFB1 intake, and the carry-over ratio was independent of AFB1 level in feed. 

Some authors reported an inverse relationship between carry-over and AFB1 intake (carry-over decreasing with intake), which could be explained by the biotransformation processes of aflatoxin in animal tissues (findings of Battacone et al. (2009) [63] with dairy ewes). Battacone et al. (2009) [63] reasoned that the contradictory results regarding the carry-over of AFB1 were explained as the gastrointestinal absorption of AFB1 and subsequent excretion, as AFM1 in milk varies among animals due to a range of influencing factors, including feeding regimens, feed digestion, animal health, hepatic biotransformation, and milk yield [63].

### 4.5. Source of Intake

Some articles highlight that the source of intake matters regarding the carry-over value. Applebaum et al. (1982) [18] studied aflatoxin transfer using 10 Holstein cows; 6 cows received pure aflatoxin for 7 days, while 3 cows received a preparation containing AFB1 and other aflatoxins and metabolites produced by an *Aspergillus* culture. One cow received a higher dose of pure aflatoxin. The effects on feed consumption and milk production were also studied during the treatment and for 5 days before and 8 days after it. While differences in total feed intake were not significant, milk production was significantly reduced and, consequently, AFM1 level was increased in the milk of cows consuming preparations containing impure (13 mg/day) AFB1 toxin. The authors hypothesized that this could be due to the effect of other metabolites produced by the mold, since the amount of AFB1 administered was the same as the amounts of pure AFB1 administered [18].

Regarding the source of contamination, Van Eijkeren et al. (2006) [56] highlighted the role the carrier of contamination played in the absorption through the gut wall. They explained that as natural contamination is expressed in terms of AFB1 equivalents and only AFB1 will be transformed to AFM1, feed composition and source type will lead to varying quantitative outcomes in different experiments and, thus, different recommendations regarding the maximum level of feed contamination leading to still-acceptable milk contamination levels [56].

Frobish et al. (1986) [14] found that the source of contamination (cottonseed meal or corn) influenced the extent of carry-over (1.73 vs. 1.32%). At the same time, AFM1 concentrations in milk were not significantly different. 

Bervis and co-authors (2021) [26] compared the AFB1 contamination level and AFM1 carry-over values of feeds from two different cow feeding systems (TMR—total mixed ration unifeed and compound feed) in 2015–2016. AFM1 occurred more frequently in the milk of cows fed compound feed. It was suggested that certain raw materials used during its production (e.g., cottonseed) were more susceptible to aflatoxin contamination [26]. They mentioned, in their article, that several studies reported lower levels of aflatoxin in the milk of animals fed by grazing than in animals fed compound feed and stored feed. The seasonal variation in the occurrence and concentration of AFM1 was likely due to the differences in the specific feeds [26].

Cottonseed was reported by other authors as well as an ingredient having higher correlation to the presence of aflatoxins in milk [48,64]. 

Hernandez and Navarro (2014) [51], on the other hand, found that wet and dry TMR (total mixed ration) feeding systems showed a higher occurrence of aflatoxins than compound feed, thus contributing the most to intakes. Aflatoxins were undetected in organic homemade compound feed samples, not showing any contribution to daily AFB1 intake. 

Walte et al. (2022) [50] hypothesized that a possible explanation for the correlation of milk yield and the aflatoxin transfer rate could be the feeding of high-yielding cows with grain-rich feeds, including a higher proportion of concentrates in the feed, which leads to decreasing pH in the rumen, being able to cause subacute ruminal acidosis. Subacute ruminal acidosis may lead to a higher absorption of AFB1 by impairing the gastrointestinal barrier function or may cause changes in the composition of the rumen microbiota and, thus, lead to alterations in the rumen metabolism. Testing this hypothesis, they could not confirm that higher proportions of concentrate feed intake lead to increased carry-over. Considering the transfer rate confirmed in that study (2.3–2.5%) and the AFB1 contamination level in feed, they assumed that the EU regulatory limit was not exceeded at that time. However, in case other sources of AFB1 are fed to cows as well as concentrates, the compliance could no longer be guaranteed [50]. 

### 4.6. Particle Size of Feed

Costamagna and coauthors (2018, 2019) [45,64] found correlations between the particle size of the feed given to animals and the carry-over of aflatoxins. AFM1 concentration in milk was higher if the particle size of the feed was deviating from the recommendation (fraction retained on the top screen exceeded 8%, meaning an excess of effective fiber in the diet). As an explanation, they observed that, in those months when the particle size distribution was not ideal, the cows made a greater selection of particles, preferably of smaller particles, causing a shorter stay time of the feed spent in the rumen, thus avoiding ruminal bacteria from degrading mycotoxins. This lower permanence of feed in the rumen could explain the higher carry-over rate found [45].

Costamagna, in her dissertation (2018) [64], reported a carry-over of 0.84–0.90% in Argentinian cows, which was also influenced by the milk production of the cows and the particle size of the feed given to them. AFM1 concentration and carry-over were higher in the milk of cows consuming feeds with inadequate particle size [64].

This parameter might have lower relevance for carry-over in studies when the whole portion is consumed by the cow, leaving no room for selection in feed. However, if consumption of the portion takes place during longer time intervals, that might still lead to an unbalanced intake resulting from selection of feed by the cows, leading to consequences for carry-over.

### 4.7. Vaccination

Vaccination is not considered here as a natural factor impacting carry-over but rather an intervention step to help reduce the carry-over experienced in regular circumstances.

Polonelli et al. (2011) [19] presented an interesting solution to the public hazard caused by milk products contaminated with aflatoxins. They reported that a vaccine based on a non-toxic modification of AFB1, anaflatoxin B1 (AnAFB1), conjugated to keyhole limpet hemocyanin (KLH), together with Freund’s adjuvant, could be effective in inducing a long-lasting titer of anti-AFB1 IgG antibodies in cows, which were cross-reactive with toxic aflatoxins including AFB1. Vaccination of lactating cows reduced the secretion of AFM1 into their milk [19]. 

In an attempt to achieve a more effective formulation, Giovati et al. (2014) [34] further studied the application injected together with various adjuvants. According to their findings, the pre-calving vaccination of heifers was potentially the most effective means of preventing aflatoxin transfer. Carry-over was reduced to 0.77% in vaccinated heifers, resulting in a 74% decrease in AFM1 concentration in milk [34].

### 4.8. Effect of Adsorbents

Similarly to vaccines in Section 4.7, the effect of interventions using adsorbents is described here shortly, as it is a promising area to tackle the challenge posed by carry-over.

A series of publications examined the potential reduction in aflatoxins in milk via the addition of certain materials (hydrated sodium calcium aluminosilicate, sodium-bentonite, yeast cell wall polysaccharides and peptidoglicanes, estherified glucomannan, sodium-montmorillonite, diatomaceous earth, activated charcoal, curcumin, etc.) as adsorbents to feed. These substances can decrease the bioavailability of the toxin by inhibiting its absorption in the intestinal tract and its delivery to the target organ. The milk, thus, contains less AFM1 compared to regular circumstances, due to the reduced absorption of AFB1 [11].

In vitro studies have reported the efficacy of several substances to bind aflatoxin B1 in feed, while experiments *in vivo* focused on the efficacy of substances in the reduction in AFB1 toxicosis in animals and the excretion of AFM1 in milk [53].

Varying levels of efficiency have been reported for activated carbons and hydrated sodium calcium aluminosilicate [29,31,35,43,65]. Regarding bentonite and its major active component, montmorillonite, most of the reviewed studies showed promising results in different study arrangements [15,20,21,32,37,38,40,49,50,66]. It is reported that clay products have not consistently prevented decreases in milk yield caused by AFB1 ingestion, and yeast fermentation products have been reported to have potential to improve animal performance parameters [42]. More recent studies have focused on the combined application of clays and yeast [17,24,42,44,45,46,67] or the use of natural compounds, particularly phytogenic compounds, such as curcumin and curcuminoids from turmeric powder [68]. A short summary of the reviewed studies is provided in Appendix A.

It can be seen from these publications that by adding adsorbents, a significant reduction in the carry-over of aflatoxins can be achieved. When evaluating the effectiveness, it is worth considering the specificity (what other, possibly essential, nutrients can the adsorbent bind other than the toxin) and the long-term effects. The effectiveness of the binding process also depends on several factors, especially the physical characteristics of the clay, e.g., particle size, total charge, and distribution [49].

Differences seen in achievement results of the cited publications may depend on details in different study arrangements. A comprehensive analysis of studies about potent aflatoxin-reducing interventions, including the use of adsorbents, was published by Farkas et al. (2022) [69].

## 5. Carry-Over Equations and Experience from Their Application

Several studies suggested mathematical formulas to describe the complex process of aflatoxin transfer from feed to milk, dependent on milk yield and other parameters. These carry-over equations are presented below, with a view on their applicability.

### 5.1. Model Established by Britzi et al. (2013) 

Britzi et al. (2013) [22] arrived at a linear, but not strong, correlation based on pairing the milk yield and carry-over values at steady state (3).

(3) $$carry-over\;\%=0.2362\times milk\;yield-4.5438,\;\mathrm{with}\;r^{2}=0.508$$

However, they found a better correlation based on exponential regression, which is described by Equation (4).

(4) $$carry-over\;\%=0.5154\;e^{0.0521}\times milk\;yield\;,\;\mathrm{with}\;r^{2}=0.6224$$

### 5.2. Model established by Pettersson et al. (1998) 

Using all the data published since 1985, including a total of 10 measurements from 5 controlled experiments, Pettersson et al. (1998) [13,70] established the following correlation (5) for the carry-over rate (based on daily intake of the animal).

(5) $$Aflatoxin\;M1\;\left(\frac{\mathrm{ng}}{\mathrm{kg}milk}\right)=10.95+0.787\times \left(µg\;aflatoxin\;B1\;intake\;per\;day\right),\;r^{2}=0.915$$

Expanding the data analysis to all studies where the daily feed contained less than 150 µg/kg AFB1 toxin (a total of 21 measurements from 6 separate studies), ignoring individual milk yields, a lower regression coefficient was observed (r^2^ = 0.417) [13].

Additionally, Pettersson and co-authors established a transfer equation earlier, in 1989 [27]. In their experiment, they investigated the degree of carry-over in the case of two groups of three cows, each yielding more than 25 kg milk a day. The value of the carry-over did not depend on the concentration of aflatoxin. On the other hand, the variability between individual animals was greater, and the transfer rate ranged between 1.7 and 3.9% (average 2.54%). The authors arrived at a linear correlation for the extent of carry-over, based on AFM1 concentration data in milk and daily intake of AFB1 (6) [27].

(6) $$Y=-0.151+0.671\times X\;(r^{2}=0.94),$$

where *Y* was ng AFM1 per kg milk and *X* was µg of AFB1 ingested per day and animal. The correlation coefficient was 0.94. 

### 5.3. Model Established by Veldman et al. (1992) 

Veldman et al. (1992) [28] established a linear relationship between carry-over and milk yield, based on their observations (7).

(7) carry−over rate=0.0013 kg milk per cow per day−0.0026, (r=0.99)

The authors stated that, despite the great individual variation, it was possible to predict the AFM1 level in milk from the AFB1 intake of a herd. There was a significant correlation between the AFM1 content of milk and the AFB1 intake, which could be expressed by the regression Equation (8).

(8) $$AFM1\;\left(\frac{\mathrm{ng}}{\mathrm{kg}milk}\right)=1.19\;AFB1\;intake\;\left(µg\;per\;cow\;per\;day\right)+1.9\;,\;(r=0.93)$$

### 5.4. Model Established by Van Eijkeren et al. (2006) 

Van Eijkeren et al. (2006) [56] described the carry-over of aflatoxins based on a mechanistic model, taking into account the research results published in the scientific literature. 

They noted that carry-over studies had generally used a small number of animals and that there was a high degree of variability in milk AFM1 levels between individual animals. The animals used in the experiments came from different breeds, concentrates with different compositions were used in the experiments, aflatoxin was added from different sources, and supplementary feeds with different compositions were used [56].

According to the model they established, part of daily consumed AFB1 is absorbed through the gut wall, metabolized into the AFM1 toxin, and eliminated via different pathways. The resulting AFM1 appears in milk and is eliminated via other routes. In the steady state of the model, the carry-over (the ratio of AFM1 excreted with milk and AFB1 ingested with feed daily) can be described, and the bioconcentration factor (the ratio of AFM1 concentration in milk and AFB1 concentration in feed) can be derived [56].

The model was analyzed by fitting it to the literature results originating from carry-over studies of different experimental designs. In spite of the expected different quantitative outcomes, similar qualitative outcomes for all experiments were reported, which could be well described by the simple steady-state model that was developed within the study of Van Eijkeren et al. (2006) [56] (9).

(9) $$C_{milk}=\frac{\alpha \times D}{\beta +M}$$

where *c_milk_* is the concentration of aflatoxin M1 in milk, *D* is the daily intake of AFB1 (µg/day), and *M* is daily milk production (kg/day), where *α* = 0.032 and *β* = 17 (based on data from Frobish et al. (1986) [14]).

The model contains two experimentally observable parameters (aflatoxin contamination and milk production rates), as well as two empirical constants (α and β), which depend on the breed of cow, the source of contamination (cottonseed, corn, groundnuts, etc.), the composition of the concentrate fed, and the composition of total feed. The different sources of AFB1, for example, have an impact on the absorption through the gut wall and will lead to different fractions of the aflatoxin mixture that will be converted into AFM1, which is further influenced by the concentrate composition and by total feed composition [56].

This was further supported by Applebaum et al. (1982) [18] who reported decreased milk yields and, consequently, increased AFM1 level in milk when naturally contaminated portions were given to the animals compared to an equivalent contamination level with pure AFB1.

The results of the different carry-over experiments can be derived from the model of Van Eijkeren et al. (2006) [56]. By fitting the model to the experimental results of Frobish et al. (1986) [14], for example, values of α = 0.032 and β = 17 were established as the empirical constants [56]. It can be derived from the model that the concentration of AFM1 in milk does not depend on the concentration of AFB1 in the feed, at constant aflatoxin intake. In addition, the carry-over value is higher for animals with higher milk production than for animals with lower milk production. It follows that the level of AFM1 is higher in cows with lower milk production than in ones with higher milk production, if the aflatoxin intake is unchanged. Carry-over does not depend on aflatoxin intake [56].

When the model was fitted to the results of the first experiment of Veldman et al. (1992) [28], there was no fit: in the first experiment by Veldman et al. (1992) [28], the AFM1 level in the milk of high-yielding cows was 1.5-times higher than that of low-yielding cows at a similar AFB1 intake, though the opposite would be expected due to a more efficient excretion route through milk [56]. The results of Veldman et al.’s (1992) [28] first experiment contradict the results of their second experiment and the results of other researchers as well. The results of their second experiment, on the other hand, fit well with the model of Van Eijkeren et al. (2006) [56].

The authors concluded that the AFB1 contamination to be achieved in connection to a given limit of AFM1 level in milk will depend on the breed of cow, the source of contamination, and total feed composition [56]. 

### 5.5. Model Established by Masoero et al. (2007) 

Masoero et al. (2007) [16] established the correlation (10) between carry-over and milk yield.

(10) $$CO\;\left(\%\right)=-0.326+0.077\times milk\;yield\left(\mathrm{kg}\right)\;(R^{2}=0.58)$$

They explained that inappropriate implementation of equations relating to milk yield and carry-over may lead to drawing erroneous conclusions regarding maximum permissible AFB1 intakes. Regarding the formula established by Veldman et al. (1992) [28], for example, they found that certain factors were not accounted for (e.g., source of contamination, variability within animals, etc.), thereby limiting their applicability for their respective trials [16]. When applying the model of Van Eijkeren et al. (2006) [56], they found as well that the model did not fit their data [16].

### 5.6. Model Established by Price et al. (1985) 

Price et al. (1985) [25] studied the carry-over of aflatoxins for 70 days in Holstein cows fed contaminated whole cottonseeds. Carry-over in the steady-state was 1.6%. The authors described the carry-over of aflatoxin into milk as follows (11).

(11) $$Y=0.0069+0.0162\times X\;(r^{2}=0.964),$$

where *Y* was µg amount of AFM1 excreted daily in the milk and *X* would be the µg amount of AFB1 consumed daily by the animal.

### 5.7. Correlation Found by Xu et al. (2021) 

Xu et al. (2021) [71] investigated the relationship of AFM1 in milk and AFB1 in TMR feeds measured in China. Applying a regression analysis on their measurements, they observed a linear relationship, which could be described by Equation (12), although P value was not significant [71].

(12) $$AFM1\;\left(\frac{\mathrm{ng}}{\mathrm{kg}}\right)=0.0456+0.0178\times AFB1\;(\frac{µg}{\mathrm{kg}})$$

### 5.8. Experience with the Carry-Over Equations

Signorini et al. (2012) [59] estimated the aflatoxin exposure from milk in Argentina using three carry-over equations published in the literature. The equation of Masoero et al. (2007) [16] determines aflatoxin transfer as a function of milk yield, while Petterson et al. (1989) [27] and Veldman et al. (1992) [28] as a function of AFB1 intake. With the equation of Veldman et al. (1992) [28], approximately twice-as-high AFM1 levels in milk were estimated, as with the equation of Masoero et al. (2007) [16].

Hernandez and Navarro (2014) [51] cited different carry-over calculations [16,28,56] and used the formula published by Van Eijkeren et al. (2006) [56] to calculate theoretical AFM levels in milk based on measured AFB1 contents in different feedstuffs. Although, the theoretical concentration ranges they calculated suggested that the likelihood of AFM1 contamination in raw cow’s milk at the studied farms was not very likely to occur, this fact could not be completely excluded in the case of changes in raw materials, intakes of concentrates, or the physiological changes that occur, particularly in high-yielding cows [51]. 

Van der Fels-Klerx et al. (2019) [72] modeled the entire production chain to study the effects of climate change on the aflatoxin B1 content of corn and the consequently appearing AFM1 level in milk. As a case study, considering the Eastern European contamination of 2013, an average Dutch milk-producing farm was studied, where the compound feed production was assumed to be based on corn imported from Eastern Europe (Ukraine). 

The complex model was built on three different climate change models, one aflatoxin B1 prediction model, and five different carry-over models [13,16,22,28,56] combined in different combinations to adequately account for uncertainty and variability, and was run 10,000 times using Monte Carlo sampling [72]. Most of the calculations predicted an increase in the aflatoxin M1 content of milk (maximum 50%) until 2030. Each combination predicted a slight increase (up to 0.6%) by 2030 in the probability of finding milk containing aflatoxin M1 above the EU limit [72].

In another piece of work, in 2016, Van der Fels-Klerx and Camenzuli (2016) [73] estimated the concentration of AFM1 in the milk of dairy cows based on different scenarios of compound feed composition, AFB1 contamination of feed, feed consumption, and milk yield. Monte Carlo simulations were used to take into account the range of possibilities in each of the steps, and the distribution of the concentration of AFM1 occurring in milk was examined through performing 1000 iterations [73].

Based on Dutch data, three different compound feed composition and two different milk yield (normal and extreme lactation) scenarios were applied in the model. Dutch monitoring data provided the AFB1 content in feed ingredients, except for a 2-week period where the extreme contamination event of 2013 was used. The carry-over of aflatoxin was modeled with the above-mentioned five different carry-over equations [16,22,28,56,70]. As an output, the ratio of AFM1 concentrations exceeding the European legal limit for milk was examined.

In all six scenarios and by taking into account any of the five carry-over equations, the ratio of modelled AFM1 concentrations exceeding the EU legal limit was not more than 1%. Considering the weeks, however, when the contaminated batch of feed was included, 28.5% of iterations exceeded the EU maximum value [73].

The results also showed that increasing milk production resulted in a minimal effect on the ratio of iterations exceeding the legal limit, which could be explained by an apparent dilution effect [73]. Under the extreme lactation scenario, even if the cows’ lactation peak was during those same weeks when they consumed the highly contaminated batch of feed, the higher carry-over rates of AFM1 did not result in greater exceedance of the regulatory level [73]. It followed that there was minimal difference in the ratios of simulations exceeding the EC limit when the scenarios of normal and extreme lactation were compared, implying that a higher milk production had an overall minimal effect on the AFM1 level in milk, despite the higher carry-over rates [73].

The probability of exceeding the limit value was higher in the case of feed with higher contamination, both for high- and low-yielding cows. On the other hand, the impact was greater in the case of the high-yield scenarios. In the case of feed with low contamination, the probability of exceeding the limit value was lower, even in cases of cows with a higher milk yield [73].

Given the same milk yield and compound feed composition, the formula describing the AFB1-AFM1 transfer played an important role in the outcome of this work. Depending on the formula used, the ratio of simulations exceeding the limit showed a difference of up to six-times. The authors described that Masoero et al. (2007) [16], Veldman et al. (1992) [28], Britzi et al. (2013) [22], and Van Eijkeren et al. (2006) [56] related the concentration of AFM1 in milk to daily milk yield and daily intake of AFB1. At the same time, the equation of Pettersson (1998) [70] set the concentration of AFM1 in milk dependent on the daily intake of AFB1 only, regardless of the daily milk yield. The carry-over rates established by Masoero et al. (2007) [16] and Van Eijkeren et al. (2006) [56] were impacted by the milk yield in the same way; therefore, similar results were obtained with these equations. However, Veldman et al.’s (1992) [28] equation generally results in a higher carry-over rate. Based on this model, the AFM1 concentration in milk would, therefore, always be the highest using the equation of Veldman et al. (1992) [28]. The equation of Britzi et al. (2013) [22] showed the strongest dependence of carry-over on milk yield, resulting in high variation in the modeled AFM1 concentration in milk, depending on the stage of lactation, while this dependence was very small based on the equation of Van Eijkeren et al. (2006) [56,73].

It was concluded from this modelling that, considering the studied compound feed compositions and AFB1 levels, the probability of the AFM1 level in milk exceeding the EU limit was very low, and only the highly contaminated feed batch could be a cause for concern, and even an increase in milk yield was not expected to increase the likelihood of exceeding the AFM1 limit in milk [73].

As regards the carry-over equations resulting in different rates of exceedance of the EC threshold, no conclusion could be drawn as to which formula was the most suitable within this model; therefore, it was suggested that all should be considered [73].

#### Transfer Model Aflatoxin B1—Dairy Cow

Based on the model of Van Eijkeren et al. (2006) [56], the “Transfer model aflatoxin B1—dairy cow, version 1.1” [74] online model was created, which, upon entering certain input data, calculates and displays the rate and time course of aflatoxin transfer. The Dutch National Institute for Public Health and the Environment (RIVM) and the Wageningen University and Research (WUR) website (https://feedfoodtransfer.nl/en), in fact, models the transfer of the toxin content of the feed into the meat, milk, or eggs of the given animal in the case of different animals and contaminants, of which a particular model examines the transfer of aflatoxins from feed into meat and milk from cows [74]. The user has to enter certain input data, such as the amount of aflatoxin contamination of the feed, the season of feeding, and the type of feeding (e.g., grass or compound feed, etc.), the daily feed consumption and milk yield, the duration of aflatoxin intake and the time elapsed afterwards, and, optionally, a limit value. The model then plots the time course of aflatoxin B1 and M1 transfer in meat, liver, kidney, and milk. A report can also be prepared from the results [74].

## 6. Carry-Over Studies Underpinning Calculations of Regulatory Limits

Knowledge of the carry-over formula may help to determine the maximum allowable contamination of the feed, which results in a given maximum milk contamination target value. In 1992, Veldman et al. [28] concluded that the average daily AFB1 intake for one cow in the herd should not reach a level of 40 µg/cow in order to keep the AFM1 level of the produced milk below the limit of 0.05 µg/kg. Therefore, a feed was considered safe in terms of AFB1 concentration if the concentration multiplied by the consumed amount gave a value below 40 µg AFB1 per day. Consequently, according to the authors, the legal limit of 5 µg/kg would not be a guarantee that the AFM1 content of the milk would be adequate [28].

The authors concluded on a maximum target value of AFB1 in feed of 3.4 µg/kg in order to keep the AFM1 level of the produced milk safe in case of high-yielding cows in early- to mid-lactation consuming 12 kg compound feed [28]. 

Britzi et al. (2013) [22] concluded that the daily AFB1 exposure of an Israeli cow in early lactation, with an average feed intake of 25 kg daily and an average milk yield of 45 kg daily, should be below 1.4 µg/kg in the feed to ensure milk with a compliant AFM1 level. 

Churchill et al. (2017) [41] applied linear regression on their experimental data to arrive at a calculated direct carry-over into milk of 6.5 µg/100 µg consumed (6.5%). The linear regression line crossed the line marking the US regulatory limit for AFM1 in milk at 15 µg/kg (ppb) aflatoxin level in feed, and this level was concluded to be the maximum likely to produce milk still below the US regulatory limits.

It can be seen from the different conclusions above that such calculations are loaded with much uncertainty, particularly because the carry-over rate itself is not a constant value but is dependent on a range of variables, including the aflatoxin intake, milk yield, or the inherent variability between the animals. 

## 7. Challenges Linked to Mycotoxin Carry-Over Research

In their review work, Völkel et al. (2011) [52] described some challenges linked to carry-over research, of which some are highlighted below. 

When evaluating literature data, and comparing different carry-over studies, experimental design needs to be considered. This includes the frequency of doses given to animals and the applied method, in addition to the dose of mycotoxins administered. Regarding contamination of the feed, it is important whether it is natural or artificial contamination; furthermore, the feed composition, the contamination of all ingredients as well as other mycotoxins and their various metabolites present in the feed need to be considered. The scale of the experiment in terms of the number of animals and the duration of exposure and the times or periods of the measurements are also important. Because of the quick excretion rate of aflatoxins, for example, it is difficult to match the exact feed material that contributed to the given milk sample. Carry-over is influenced by a range of parameters, including the animal’s species, breed, sex, age, and milk production level, in addition to the general health status of the animal; therefore, all these factors need to be taken into account.

In many cases, the different studies cannot be compared due to the different study arrangements, and the parameters for calculating the carry-over are not standardized [52]. Regarding breed, for instance, Britzi et al. (2013) [22] used Israeli Holstein cows, while Masoero et al. (2007) [16] carried out their study on Holstein cows, and Veldman et al. (1992) [28] did not specify the breed. Concerning the contaminated feed applied in the studies, Britzi et al. (2013) [22] and Masoero et al. (2007) [16] used contaminated corn meal, while Veldman et al. (1992) [28] used contaminated groundnut meal, and the model established by Van Eijkeren et al. (2006) [56] was fitted to the results of Frobish et al. (1986) [14] using contaminated cottonseed. 

The established carry-over values varied accordingly. Studies should follow similar experimental designs in order to be more comparable. 

The great variation in carry-over values from different studies can also be partly explained by analytical considerations, such as the different LOD (limit of detection) values of the applied methods. High LOD values can result in false-negative values. The homogeneity achieved during sample preparation is also of fundamental importance, as in the case of mycotoxins, the tested substance may be present in the sample in clusters. In addition, the concept of “masked mycotoxins” is an important aspect. These substances are metabolites of the concerned mycotoxins, which can be formed in the plant or fungus, e.g., by conjugation with polar compounds. Due to their difficult detection, their presence is a special problem when using naturally contaminated feed ingredients. On the one hand, the specific amount of the applied mycotoxin dose is incorrect, and on the other hand, these substances can contribute to the background contamination. In addition to the mycotoxin concerned, naturally contaminated feed may also contain its various metabolites, which further complicates the assessment. This creates uncertainties in the analysis of relationships between mycotoxin levels in feed and their effective doses within the body [52]. Miklós et al. (2020) [75] summarized the analytical methods suitable for the determination of aflatoxins in different matrices.

## 8. Directions for Future Carry-Over Research 

Climate change and extreme weather conditions are increasingly taking place, which must be considered in the context of food safety challenges. Both unexpected mycotoxins and unusual contaminant levels may occur in the future. As the extent of carry-over depends largely on the animal’s health status, health changes seen during daily examinations (liver damage, mastitis, ruminal acidosis) should also be considered in carry-over research [52]. 

The fact that different mycotoxins can be detected side by side in different crops makes it necessary to investigate their combined (synergistic, cumulative, or antagonistic) effects [52]. All these directions may determine future research on the carry-over of aflatoxins.

## 9. Final Discussion

According to the published literature, the carry-over of aflatoxins may be affected by several factors in connection to feed, the animal, or other aspects. The extent of carry-over can be estimated by regression on the measured data points or by describing the process using a mechanistic model. The carry-over equations estimated the transfer rate into milk depending on two main factors, milk yield and the intake of aflatoxin. Van Eijkeren et al. (2006) [56] included two additional empirical constants in their model, which depend on the breed of cow, the source of contamination (cottonseed, corn, groundnut, etc.), the composition of the concentrate fed, and the composition of total feed. The so-called Transfer model aflatoxin B1—dairy cow model [74]—building on the model of Van Eijkeren et al. 2006 [56], requires the contamination level of feed, the season of feeding, the type of feed, feed consumption, and milk yield as inputs. These factors are important determinants of the actual carry-over value. This study investigated some factors in detail, which are summarized below. 

One of the most important factors is milk yield (daily milk production). Generally, increasing milk yield results in increasing carry-over. Milk yield can be influenced by the lactation stage but also by the breed of the animal (e.g., Holstein cows are primarily bred for milk production). Van Eijkeren et al. (2006) [56] assumed a linear correlation between milk production and the amount of feed consumed in a given range of daily milk production, meaning the more the animal consumes, the more milk will be secreted, which also affects the transfer rate of aflatoxins. The number of daily milkings also contributes to the increase in milk yield. According to Britzi et al. (2013) [22] and Churchill (2017) [41], for example, intensive large-scale production was characterized by milking three times a day.

An increase in somatic cell count occurs in mastitis, which is associated with an increase in membrane permeability, presumably resulting in an increase in aflatoxin transfer. However, research has not found a correlation between somatic cell count and aflatoxin carry-over per se [16,22,45].

The seasonal changes clearly influence the aflatoxin contamination that occurs during the cultivation of crops intended for animal feed. In addition, seasons can also affect the feeding itself, because in periods suitable for grazing, the animals can consume feed consisting of fewer stored raw materials in addition to more fresh produce. In the case of stored products, greater aflatoxin contamination occurred [26,54]. 

An increase in aflatoxin contamination of feed under unchanged conditions results in a higher aflatoxin intake and, in absolute terms, more aflatoxin excreted in milk. Contamination depends on the type of feed. Greater and more frequent aflatoxin contamination in milk was reported when compound feeds were consumed [26] and certain raw materials (e.g., cottonseed) were presumed to be more susceptible to contamination with aflatoxin [26]. The study of Van der Fels-Klerx and Camenzuli (2016) [73] showed that an increased use of maize in compound feed, combined with an increased contamination level of maize with AFB1, might increase the probability of exceedance of the EC limit for AFM1 in milk.

According to certain research, deviation in the particle size of feed from the ideal level may also increase the carry-over of aflatoxins by enabling the animals to select the parts from the feed that can be digested more quickly, thereby shortening the time available for the breakdown of toxins in the rumen [45].

In addition to natural factors affecting the carry-over, several intervention steps may help, with good results in reducing the amount of aflatoxins in the milk. In this context, the effects of vaccination and the use of adsorbents were mentioned. 

The discussed factors determining the carry-over and their further correlations are summarized in Table 3.

## 10. Conclusions

Our aim with this review was to extract and synthesize information from the published literature regarding the process and extent of carry-over of AFB1 from feed into AFM1 in cow milk, and to review the most important factors influencing this process.

It can be seen from Table 3 that the factors affecting carry-over of aflatoxins may have further correlations with each other and with other factors. The most important factors identified and discussed were milk yield, somatic cell counts, aflatoxin B1 intake, source of contamination, seasonal effects, particle size of feed, and the effects of certain interventions, namely vaccination and the use of adsorbents. Carry-over equations provide further information regarding the relationship of transfer rate and, particularly, two factors, milk yield and aflatoxin B1 intake. Summarizing different study results is difficult, among others, due to differences in study arrangements and differences in milking and feeding conditions of animals. The different carry-over equations may lead to largely different results, and no single carry-over equation can be picked as the best one. Further carry-over research should attempt to record as many influencing circumstances as possible in order to properly evaluate and compare the results.

## 11. Methodology

The goal of this study was to describe the extent of the conversion of aflatoxin B1 consumed by the cow with feed into aflatoxin M1 in milk. 

The Scopus database was searched for relevant articles regarding carry-over, first in May 2022 and checked again in January 2023, with the keywords (in “Article title, Abstract, Keywords”): aflatoxin AND (“carry-over” OR “carry over” OR carryover OR transfer OR conversion) AND (milk OR dairy), resulting in 175 publications, of which 97 were deemed relevant by their titles.

Species other than cows and milk products other than milk were not the focus of this assessment. Some of these publications are mentioned in our paper; however, these articles are not included in the strict analysis of papers. Similarly, papers dealing with only aflatoxin contamination of milk were only included if they contained relevant information regarding factors affecting carry-over as well. 

The records were screened by their abstracts, leaving 75 articles for full-text reading and extraction. Based on full-text reading, 55 articles were left for processing.

In addition, 16 articles referenced by these publications deemed relevant for the subject were also included (snowball articles).

Eight factors, milk yield, somatic cell counts, seasonal effects, aflatoxin B1 level in feed and intake, source of intake, particle size of feed, vaccination, and adsorbents, were selected for further consideration based on first reading. Studies discussing these factors are plotted in Appendix B. During second reading, facts regarding these effects, and numerical and mathematical formulae regarding the extent of carry-over, were extracted from the papers and summarized in our study. 

## Figures and Tables

**Table 1:** Times reported for aflatoxin M1 peak and elimination from milk.

AFM1 Maximum in Milk	AFM1 Elimination Time from Milk	Source
5–7 days	4 days	Applebaum et al. (1982), [18]
24 h	24 h	Frobish et al. (1986), [14]
3 days	4 days	Diaz et al. (2004), [15]
5 days		Masoero et al. (2007), [16]
7 days	5 days	Polonelli et al. (2011), [19]
24 h	5 days	Sumantri et al. (2012), [20]
	3 days	Queiroz et al. (2012), [21]
3 days	below 0.05 μg/kg after 1 day	Britzi et al. (2013), [22]
	3 days	Xiong et al. (2015), [17]
4 days	4 days	Guo et al. (2019), [23]
24 h	max 2 days	Guo et al. (2021), [1]
	5 days	Cha et al. (2021), [24]

**Table 2:** Carry-over rates.

Carry-Over (%)	Publication	Comment
2.64	Patterson et al. (1980), [2]	
1.6	Price et al. (1985), [25]	
1.74	Frobish et al. (1986), [14]	
2.54	Pettersson et al. (1989), [27]	
6.2	Veldman et al. (1992), [28]	at the beginning of milk production
1.8	Veldman et al. (1992), [28]	at the end of milk production
3.8	Veldman et al. (1992), [28]	high-yielding cows
2.5	Veldman et al. (1992), [28]	low-yielding cows
0.45–0.55	Galvano et al. (1996), [29]	without adsorbent
0.15–1.31	Choudhary et al. (1998), [30]	
2.25	Diaz et al. (2004), [15]	without adsorbent
1–2	EFSA (2004), [13]	cows with low milk production
6	EFSA (2004), [13]	cows with high milk production
2.32	Masoero et al. (2007), [16]	high yield, high somatic cell count
2.70	Masoero et al. (2007), [16]	high yield, low somatic cell count
1.48	Masoero et al. (2007), [16]	low yield, high somatic cell count
1.29	Masoero et al. (2007), [16]	low yield, low somatic cell count
2.65	Kutz et al. (2009), [31]	without adsorbent
3.85	Pietri et al. (2009), [32]	without adsorbent
2.35	Bantaokul and Ruangwises (2010), [33]	
0.1	Sumantri et al. (2012), [20]	without adsorbent
0.61	Queiroz et al. (2012), [21]	without adsorbent
2.50	Britzi et al. (2013), [22]	cows in later stages of lactation
5.80	Britzi etal. (2013), [22]	cows in middle stage of lactation
3.40	Giovati et al. (2014), [34]	
1.8	Rojo et al. (2014), [35]	without adsorbent
0.56	Xiong et al. (2015), [17]	without adsorbent
0.22–3.74	Dimitrieska-Stojkovic et al. (2016), [36]	
1.07	Maki et al. (2016), [37]	without adsorbent
1.17	Soufiani et al. (2016), [38]	without adsorbent
6.5	Churchill et al. (2016), [39]	
2.737	Korgaonkar et al. (2017), [8]	without adsorbent
1.37	Sulzberger et al. (2017), [40]	without adsorbent
6.5	Churchill (2017), [41]	
1.65	Jiang et al. (2018), [42]	without adsorbent
0.45	Pate et al. (2018), [43]	without adsorbent
1.38	Xiong et al. (2018), [44]	without adsorbent
0.84 (0.05–5.93)	Costamagna et al. (2019), [45]	average carry-over
1.21 (0.23–5.93)	Costamagna et al. (2019), [45]	high-yielding cows
0.48 (0.05–2.12)	Costamagna et al. (2019), [45]	low-yielding cows
2.7	Rodrigues et al. (2019), [46]	without adsorbent
0.6–6	Rodríguez-Blanco et al. (2020), [47]	
3.22	Bervis et al. (2021), [26]	
1.16	Cha et al. (2021), [24]	without adsorbent
0.70 (0.02–7.3)	Costamagna et al. (2021), [48]	
1.15–2.30	Guo et al. (2021), [1]	
0.52	Hajmohammadi et al. (2021), [49]	without adsorbent
2.3–2.5	Walte et al. (2022), [50]	without adsorbent

**Table 3:** Factors affecting the carry-over of aflatoxins.

Factor	Influence on Carry-Over	Sub-Effects	Other Influencing Factors	Remarks
Daily milk production (milk yield)	↑		time passed after calving (stage of lactation)↓; daily milkings↑; breed↕; feed consumption↑;	animal husbandry related
Somatic cell count	not confirmed	(permeability in the udder↑)		animal related, would be relevant only for very high SCC cases
Season-effect	↕	grazing↓; stored feed ingredients↑; warm, humid climate↑		other factor
AFB1 intake	↑			
Effect of feed (contamination source)	↕	feed contamination↑; compound feed↑; ‘’susceptible ingredients”↑		feed related
Feed particle size (different from ideal)	↑			feed related
Vaccination	↓			animal husbandry related intervention
Adsorbents	↓			feed related intervention

↑: increase; ↓: decrease.

## Data Availability

No new data were created or analyzed in this study. Data sharing is not applicable to this manuscript.

## Appendix A. Carry-Over Studies with Adsorbents

### Appendix A.1. Activated Carbons and HSCAS

Galvano et al. (1996) [29] studied the effect of using two activated charcoals, CAC1 and CAC2, and hydrated sodium calcium aluminosilicate (HSCAS) on the aflatoxin carry-over values in 12 Friesian cows in late lactation. The applied adsorbents were effective. When adding CAC1, the AFM1 concentration in milk was reduced to a higher extent (45.3%) than with HSCAS (32.5%). On the other hand, CAC2 reduced AFM1 content in milk by only 22%. Pelleting was unsuccessful in the case of CAC2, which could explain the observed lower performance of CAC2.

Carry-over ranged between 0.45 and 0.55% during the first and third weeks (when no adsorbents were added), while in the second week, when adsorbents were added, between 0.27 and 0.41%. The carry-over values related to milk yield were lower compared to the values observed by other authors [29].

The AFM1 content in milk was significantly lower in the second week (when adsorbents were added), compared to the others. Omitting the adsorbent from feed during the third week resulted in the aflatoxin content in milk returning to at least the same level as in the first week. AFM1 secretion, on the other hand, remained constant during the 3 weeks due to decreasing milk yield. The authors noted that average AFB1 intake was higher (55 µg/day) than the 40 µg/day maximum intake established by Veldman et al. (1992) [28], which was calculated to result in acceptable AFM1 contents in milk; however, the contamination level of milk did not reach the regulatory maximum level set by EU.

Later publications mention the variable effectiveness of activated carbons. The reason for this may be that the specificity of activated carbon is relatively low, and it also binds essential nutrients in addition to mycotoxins [9].

Using HSCAS, on the other hand, Harvey et al. (1991) [65] achieved a 24–44% reduction in AFM1 levels in milk, depending on the level of aflatoxin in the feed and the concentration of the adsorbent added. Rojo et al. 2014 [35] and Pate et al. 2018 [43] also experienced a reduction in AFM1 transfer when aluminosilicate adsorbents were used.

Kutz et al. 2009 [31] also observed a reduction in AFM1 transfer via the addition of two HSCASs, NovasilPlus and Solis, while the addition of MTB-100, a preparation including modified yeast cell culture and HSCAS, was not effective. In contrast to this, Diaz et al. 2004 [15] experienced a 59% reduction in AFM1 concentrations due to the addition of MTB-100.

### Appendix A.2. Bentonites, Montmorillonite

Montmorillonite is a major active component of bentonite. A range of studies examined the effects of adding this clay into cow feed on the transfer of mycotoxins into milk.

Veldman (1992) [66] added bentonite to the feed of 24 cows. The adsorbent reduced the level of AFM1 in their milk by 1/3. The effects of bentonite and aluminosilicate were compared in a second study with a similar experimental design, where bentonite reduced carry-over by one-third as well. However, no effects were observed when aluminosilicate was used [66].

Diaz et al. (2004) [15], based on their previous *in vitro* results, studied the aflatoxin binding capacity of six materials. They found that carry-over values were reduced by 61%, 65%, and 50% for sodium-bentonites, 59% for an esterified glucomannan (MTB-100), and 31% for calcium-bentonite, while activated carbon added at 0.25% had no effect.

Pietri et al. (2009) [32] investigated the effect of bentonite-based Mycofix^®^ Plus (MPL) adsorbent on the transfer of aflatoxin into cow’s milk. Adding a small amount of MPL (20 g, 0.047%) reduced aflatoxin transfer by 31%, while a large amount (50 g, 0.12%) of MPL by 41%. These results were promising as the applied levels of the adsorbent were much lower compared to other studies [32].

Studying the effect of treatment with two doses of montmorillonite-based adsorbent, Queiroz et al. (2012) [21] found that treatment with the higher dose (1%) reduced the AFM1 content in milk (by 17%) and the toxin was eliminated more quickly. No toxin could be detected in milk after 1 day.

In their 2012 experiment, Sumantri et al. (2012) [20] observed no effect from the addition of bentonite on aflatoxin level in milk. The authors argued that in vitro binding capacity of AFB1 adsorbents had not always been comparable to in vivo responses. The efficacy of adsorbents was affected by a range of parameters, e.g., bentonite could bind 100% of AFB1 present in aqueous solution [20].

Maki et al. (2016) [37] studied the effect of NovaSil Plus, a stated calcium montmorillonite, and found a significant reduction in AF transfer rates at two concentrations added to contaminated feed. Sulzberger et al. (2017) [40] found that an adsorbent composed of vermiculite, nontronite, and montmorillonite, added in different concentrations, linearly reduced the aflatoxin transfer from the rumen into the milk.

Soufiani et al. (2016) [38] compared the effects of natural and processed bentonite (local or commercially available) and found that the aflatoxin contents in milk and transfer rates were decreased only by using locally processed bentonite. They concluded that processing bentonites by means of entering sodium ions into the interlayer space of the clay could considerably increase the adsorption of aflatoxin from feed and decrease aflatoxin transfer into milk.

Hajmohammadi et al. (2021) [49] studied the effect of bentonite with a given size distribution (<5 microns) on aflatoxin transfer, in comparison with the effect of a commercially available adsorbent. It was found that the effects of the two adsorbents were comparable and resulted in a decrease in the carry-over value (0.43 and 0.40% compared to 0.52%).

In one of the latest studies on adsorbents, Walte et al. (2022) [50] also tested a bentonite-montmorillonite-based adsorbent, which led to a strong reduction in AFM1 concentration of milk.

### Appendix A.3. Addressing Potential Side Effects of Clay-Combined Addition of Clay and Yeast or Natural Compounds

Considering that clay products have not consistently prevented decreases in milk yield caused by AFB1 ingestion [42], later studies on the effects of adsorbents focus on combined applications, including clay and yeast. *Saccharomyces cerevisiae* fermentation products (SCFPs) were reported to have potential to improve animal performance parameters [42].

Jiang et al. (2018) [42] studied the effects of a bentonite clay added to the diet of cows, with or without a high level of *Saccharomyces cerevisiae* fermentation product, and found that both treatments reduced the AFM1 concentration in milk and the transfer of AFB1 into milk AFM1 (1.01% and 0.94% compared to 1.65%). Although the carry-over decreasing effect was comparable between the two treatments using clay with and without *Saccharomyces cerevisiae* fermentation product, the authors found that adding the yeast fermentation product along with clay was better at maintaining milk production during an aflatoxin challenge than using the clay alone [42].

On the other hand, Weatherly et al. (2018) [67] found no differences in AF transfer or excretion in milk, and the adsorbent neither hindered nor improved cow feed intake, milk production, or efficiency, when yeast cell wall and bentonite-based adsorbent were studied together.

Xiong et al. (2015) [17], using Solis Mos, an adsorbent containing sodium montmorillonite with live yeast on cows in later stages of milk production, consuming low or high aflatoxin levels, found that in addition to improving the antioxidant status independently of aflatoxin intake, the addition of the adsorbent eliminated the AFM1 background contamination of milk in the case of animals consuming uncontaminated feed as well. AFM1 concentration in milk and carry-over were reduced by 16% and 17.9%, respectively, when the adsorbent was given to the animals consuming low amounts of aflatoxin. No change in AFM1 excretion caused by the adsorbent was observed in the case of animals consuming larger amounts of aflatoxins [17].

Testing the same adsorbent during a long-term aflatoxin challenge, Xiong et al. (2018) [44] observed a significant reduction in milk AFM1 concentrations and transfer rates.

Rodrigues et al. (2019) [46] tested the efficacy of two additives composed of high adsorbent clay minerals and inactivated yeast, Toxy-Nil and Unike Plus, and found that both adsorbents reduced the AF transfer into milk significantly. Supplementation with these also reduced the time required for the AFM1 level in milk to drop below the FDA limit after AFB1 was withdrawn from the diet [46].

Cha et al. (2021) [24] experienced significantly lower milk AFM1 concentrations and AFB1-AFM1 transfer rates (0.57% and 0.63% compared to 1.16%) when a montmorillonite-diatomite based patented adsorbent and a montmorillonite, diatomite, yeast cell wall and sodium-alginate-based adsorbent were fed to cows in addition to an aflatoxin challenge.

In one of the most recent works, Girolami et al. (2022) [68] studied the effects of curcumin and curcuminoids from turmeric powder on the carry-over of aflatoxins. Beneficial effects had previously been demonstrated with this substance *in vitro* and in other food-producing species, including ruminants. However, adding turmeric powder to the diet did not substantially affect the AFM1 content in dairy milk, which, according to the authors, could be due to the limited bioavailability of the formulation and warranted further investigations [68].

## Appendix B. Publications Examining Different Effects on Carry-Over

**Table A1:** Publications examining different effects on carry-over.

Author	Milk Yield	SCC	AFB1 Level/Intake	Source	Particle Size	Season	Vaccination	Adsorbents
Applebaum et al. (1982), [18]				x				
Bervis et al. (2021), [26]				x		x		
Britzi et al. (2013), [22]	x	x						
Cha et al. (2021), [24]								x
Churchill (2017), [41]	x		x					
Churchill et al. (2016), [39]	x		x					
Costamagna (2018), [64]	x				x	x		
Costamagna et al. (2019), [45]	x	x			x	x		
Costamagna et al. (2021), [48]	x				x	x		
Diaz et al. (2004), [15]								x
Frobish et al. (1986), [14]	x		x	x				
Galvano et al. (1996), [29]								x
Giovati et al. (2014), [34]							x	
Girolami et al. (2022), [68]								x
Gómez (2008), [58]						x		
Hajmohammadi et al. (2021), [49]								x
Hernandez-Martinez and Navarro (2014), [51]				x		x		
Jiang et al. (2018), [42]								x
Khaneghahi Abyaneh et al. (2019), [61]						x		
Korgaonkar et al. (2017), [8]								x
Kutz et al. (2009), [31]								x
Lafont et al. (1980), [57]		x						
Maki et al. (2016), [37]								x
Masoero et al. (2007), [16]	x	x						
Pate et al. (2018), [43]								x
Pena-Rodas et al. (2018) [62]						x		
Pettersson et al. (1989), [27]			x					
Pietri et al. (2009), [32]								x
Polonelli et al. (2011), [19]							x	
Price et al. (1985), [25]			x					
Queiroz et al. (2012), [21]								x
Rodrigues et al. (2019), [46]								x
Rojo et al. (2014), [35]								x
Schirone et al. (2015), [60]						x		
Signorini et al. (2012), [59]			x			x		
Soufiani et al. (2016), [38]								x
Sulzberger et al. (2017), [40]								x
Sumantri et al. (2012), [20]			x					x
Van der Fels-Klerx and Camenzuli (2016), [73]			x					
Van der Fels-Klerx et al. (2019), [72]			x					
Van Eijkeren et al. (2006), [56]	x		x	x				
Veldman et al. (1992), [28]	x		x					
Walte et al. (2022), [50]				x				x
Weatherly et al. (2018), [67]								x
Xiong et al. (2015), [17]								x
Xiong et al. (2018), [44]								x
Xu et al. (2021), [71]			x					
